# Snapping Shoulder Secondary to Subluxation of an Accessory Coracobrachialis

**DOI:** 10.7759/cureus.7967

**Published:** 2020-05-05

**Authors:** Hardi Madani, Sarim Ather, Roxanne Giggens, Hira Hasan, David McKean

**Affiliations:** 1 Radiology, East and North Hertfordshire NHS Trust, Stevenage, GBR; 2 Radiology, Oxford University Hospitals NHS Foundation Trust, Oxford, GBR; 3 Radiology, Cambridge University Hospitals NHS Foundation Trust, Cambridge, GBR; 4 Radiology, Buckinghamshire Healthcare NHS Trust, Aylesbury, GBR

**Keywords:** shoulder, anatomy, accessory, muscle, us, mri, coracobrachialis

## Abstract

Numerous snapping syndromes have been reported in the musculoskeletal system. Identifying the cause of these symptoms can often be challenging as the underlying abnormality may not be appreciable on routine static examinations. We report a 30-year-old female who presented with an unusual snapping sensation in her right anterior shoulder. This was readily reproducible during shoulder abduction with a palpable clicking evident on clinical examination. Dynamic ultrasound revealed this to be secondary to an accessory coracobrachialis muscle, which subluxed suddenly over the anterior subscapularis tendon during abduction. An accessory coracobrachialis muscle is a rare normal variant that is often asymptomatic. Extra-articular causes of shoulder snapping have been rarely reported, and this is the first case report of an accessory coracobrachialis muscle causing a snapping shoulder phenomenon.

## Introduction

Snapping phenomena have been reported to occur in a wide variety of regions of the body and are usually related to abnormal movement of soft structures in close relation to a joint [1]. Snapping sensations are common in the general population; however, in some cases these snapping phenomena can be debilitating and associated with significant pain [1,2]. Diagnosis of the underlying cause of these snapping sensations can be challenging as static images do not demonstrate the abnormal movement of the anatomical structures causing these symptoms. Ultrasound is a dynamic test that allows for real-time assessment of the movement of the underlying anatomical structures. However, there are still a number of limitations to this technique, including being dependent on the patient being able to reproduce the snapping sensation, the limited range of ultrasound to visualise deep structures and variability in the skill and experience of the operator performing the examination [1,2]. 

Snapping phenomenon have been described in the anterior hip secondary to the rotational movement of the iliopsoas tendon, lateral hip due to snapping of the iliotibial band over the greater trochanter and posterior hip due to snapping of the long head of the biceps femoris against the ischial tuberosity [3-5]. Snapping sensation in the knee has been reported due to a variety of intra- and extra-articular causes, including intra-articular bodies, plica and meniscal tears [1]. Ankle snapping is often associated with instability of the ankle tendons within the retromalleolar grooves [6]. Snapping of the elbow may be extra-articular, most commonly related to anterior dislocation of the ulnar nerve or the distal end of the medial triceps above the medial epicondyle, or intra-articular, when it may be related to the synovial fringe capsulosynovial layer at the junction between the radial collateral ligament and the annular ligament [7,8]. Snapping sensation in the wrist has been classically described due to underlying carpal instability or tendon instability such snapping of the extensor carpi ulnaris secondary to a subsheath tear [9,10].

Snapping in the shoulder region has previously been reported secondary to “grating” of the scapula caused by impingement between the medial border of the scapula and the adjacent ribs [11]. However, relatively few other causes of extra-articular snapping at the shoulder joint have been previous published.

## Case presentation

**Figure 1:**
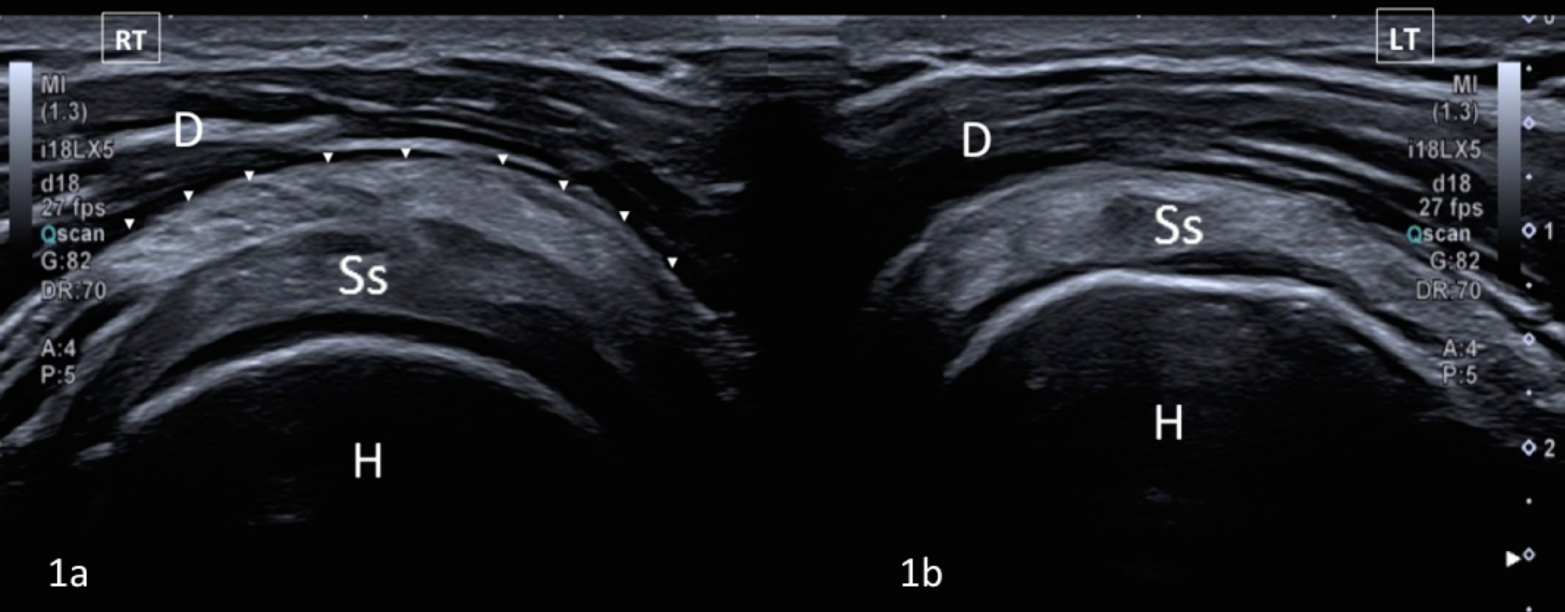
Transverse ultrasound of the anterior shoulder performed using a 15-MHz linear array ultrasound transducer (Aplio i700, Cannon, UK). (a) An image demonstrating an accessory coracobrachialis muscle (arrowheads) in close relation to the subscapularis tendon (Ss) and the deltoid muscle (D). Humeral head indicated by (H). (b) A comparative transverse ultrasound image of the left shoulder of the same patient and demonstrates no accessory coracobrachialis muscle.

**Figure 2:**
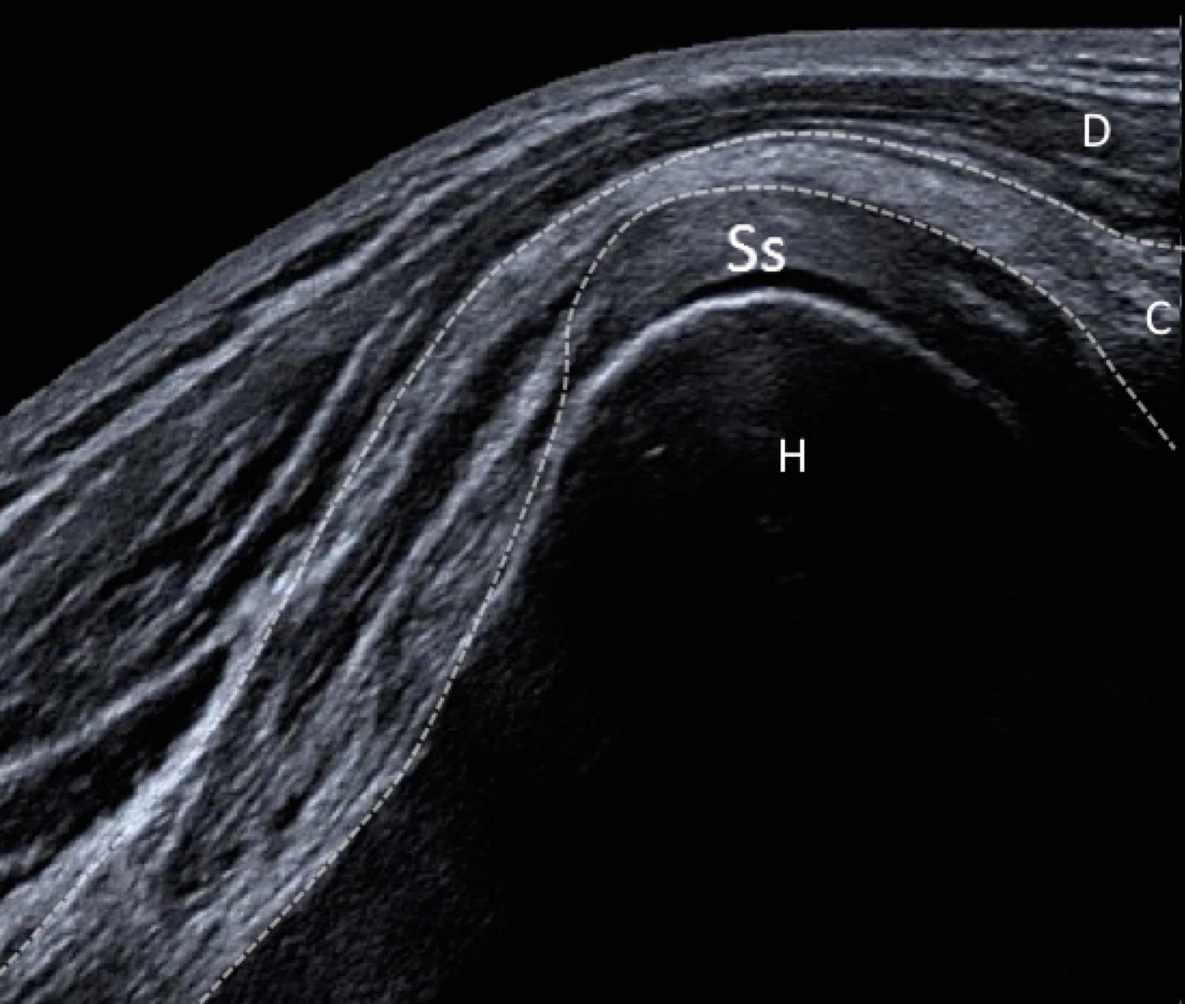
Panoramic longitudinal ultrasound acquired using a 15-MHz linear array ultrasound transducer (Aplio i700, Cannon, UK) demonstrates the accessory coracobrachialis muscle (dashed line) arising from the coracoid process (C), overlying the subscapularis tendon (Ss), deep to the deltoid muscle. Humeral head indicated by (H).

A 30-year-old female patient presented with a history of many years of a snapping phenomenon over the anterior aspect of her right shoulder. She had no other relevant medical history, a normal chest x-ray and was referred for dynamic ultrasound to identify the cause of these symptoms. A palpable clicking sensation could be elicited during physical examination on abduction of the glenohumeral joint. Ultrasound demonstrated an abnormal accessory muscle arising from the apex of the coracoid process lying superficial to the subscapularis muscle which was identified as an accessory coracobrachialis muscle (Figures 1, 2).

During real-time dynamic imaging, a sudden jerky subluxation of the accessory coracobrachialis muscle was demonstrated which corresponded with the palpable click and snapping sensation reported by the patient (Video 1).

**Video 1:** Dynamic ultrasound performed during lateral abduction rotation of the right glenohumeral joint demonstrating sudden subluxation of the accessory coracobrachialis muscle over the subscapularis tendon which corresponded with the characteristic snapping symptoms reported by the patient.

**Figure 3:**
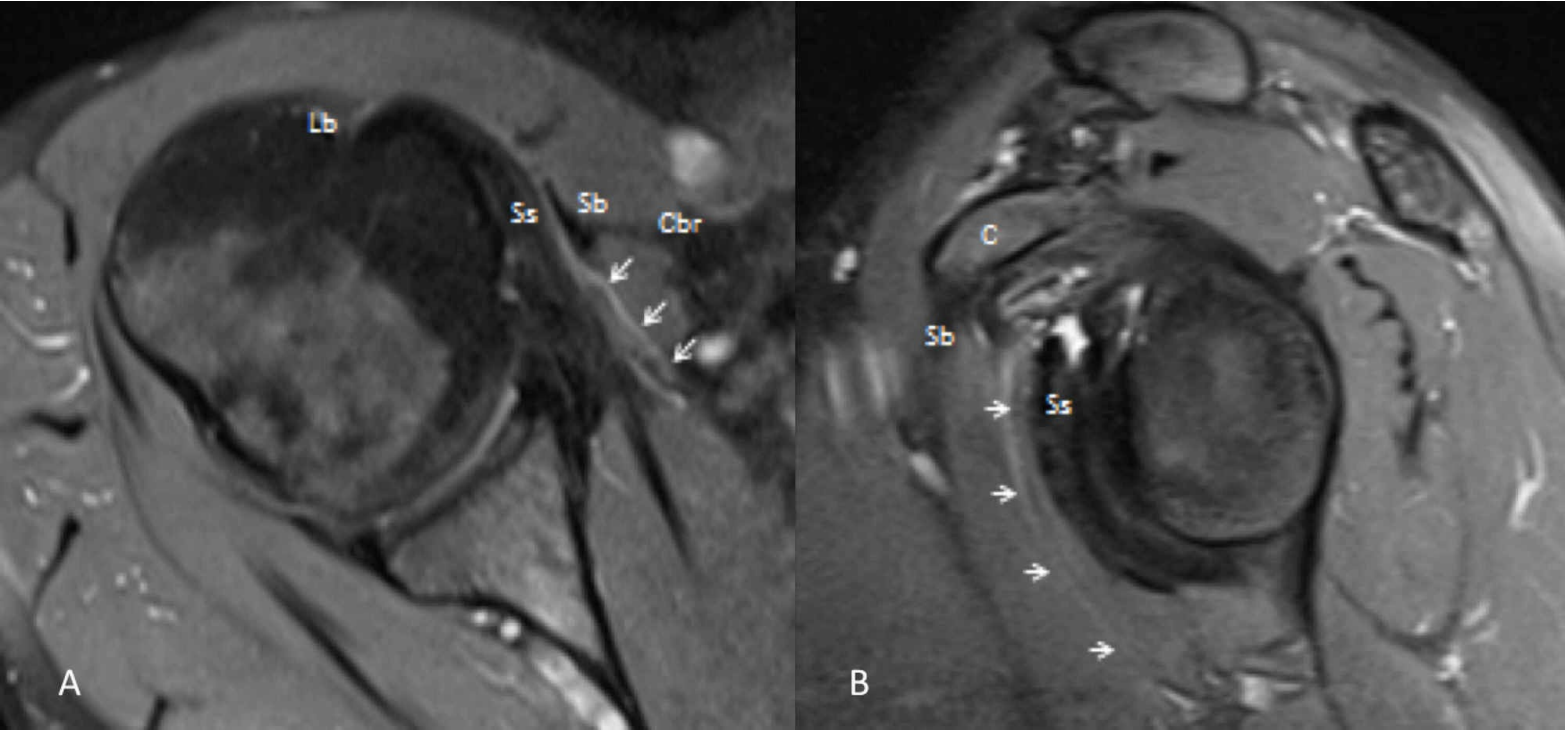
Axial (A) and sagittal oblique (B) proton-density fat-suppressed weighted images of the accessory coracobrachialis muscle (arrows). Ss subscapularis, Lb long head biceps brachii, Sb short head biceps brachii, CBr coracobrachialis.

Subsequent shoulder MRI confirmed the presence of a small accessory coracobrachialis muscle (Figure 3). 

A diagnostic/therapeutic ultrasound-guided steroid injection was performed to confirm that the accessory muscle was the source of patient symptoms, and a solution of 40 mg triamcinolone acetonide and 3 ml 0.25% bupivacaine was instilled between the accessory coracobrachialis and subscapularis muscle. Unfortunately, symptomatic relief was only temporary and the patient has declined any further intervention.

## Discussion

An accessory coracobrachialis muscle is a rarely seen anatomical variant that may be identified on shoulder ultrasound with a reported prevalence of 1.04% [12]. The coracobrachialis muscle classically originates from the apex of the coracoid process, together with the short head of the biceps brachii, and inserts on the medial surface of the shaft of the humerus between the attachments of the triceps and brachialis. This muscle flexes the arm medially and abducts. In most species, the coracobrachialis has three portions: the coracobrachialis longus, medius and brevis. In humans, however, the medius and longus fuse to form the coracobrachialis muscle [13].

In this case, the accessory coracobrachialis muscle, also known as the coracobrachialis brevis, arose from the inferior margin of the coracoid process and ran obliquely across the anterior surface of the subscapularis tendon before inserting onto the humeral neck [14]. While usually asymptomatic, it has been reported that an accessory coracobrachialis muscle may have a compressive effect and result entrapment of adjacent neurovascular structures, such as the musculocutaneous nerve, median nerve or the lateral cord of the brachial plexus [15]. However, no neurological symptoms were present in the patient. Restriction of external rotation and subcoracoid impingement have also been reported [16,17].

Extra-articular causes of shoulder snapping are relatively rare. Painful anterior shoulder snapping provoked by shoulder extension with the shoulder abducted has been described secondary to impingement between the coracoid process and subcoracoid bursitis [18]. One case report exists of an ectopic insertion of the pectoralis minor tendon causing a clicking sensation in the shoulder [19]. A locking sensation has also been described due to subacromial impingement of a tendinous flap of a longitudinally torn supraspinatus tendon on shoulder abduction [20]. However, to the best of our knowledge this is the first case of a shoulder snapping phenomenon to be reported associated with an accessory coracobrachialis muscle.

## Conclusions

Snapping secondary to the abrupt movement of anatomical structures may be found in a wide variety of locations. Identifying the underlying pathology may be challenging. In this case, dynamic sonography allowed for real-time demonstration of sudden abnormal subluxation of an accessory coracobrachialis muscle over the subscapularis tendon resulting in a palpable painful snap during abduction of the glenohumeral joint. Musculoskeletal radiologists should be aware of this rare potential cause of anterior shoulder snapping.
